# Healing Process of Rat Skin Wounds Treated With Vitamin C and Low-Intensity Laser Therapy

**DOI:** 10.7759/cureus.11933

**Published:** 2020-12-06

**Authors:** Luis Flavio Duraes Gomes Oliva, Doroty Mesquita Dourado

**Affiliations:** 1 Medicine, Anhanguera-Uniderp University, Campo Grande, BRA; 2 Biological Sciences, Anhanguera-Uniderp University, Campo Grande, BRA

**Keywords:** wound healing, low-intensity laser therapy, vitamin c, collagen

## Abstract

Introduction: This study evaluated, from a histological point of view, the process of repairing skin wounds caused in the dorsal region of rats when subjected to treatment with vitamin C, low-intensity laser, and association of both.

Methods: Forty-eight adult male rats (*Rattus norvegicus*, albinus, Wistar), weighing between 250 and 300 g were used in this study. The rats were anesthetized with sodium pentobarbital (10 mg/kg) intraperitoneally (IP) and a circular area of ​​skin of approximately 8 mm in diameter was removed from the dorsal region of their back by a punch. The animals were randomly divided into four groups of 12 individuals: Group I, control group, was treated with saline solution; Group II was treated with topical application of vitamin C; Group III was treated with low-intensity laser; and Group IV was treated with both low-intensity laser and topical application of vitamin C. Samples were histologically analyzed through optical microscopy with hematoxylin and eosin staining and collagen I and III concentrations were quantified using the picrosirius-hematoxylin histochemical method and further submitted to statistical analysis.

Results: Whilst the control and vitamin C groups admittedly showed slight epithelial proliferation at the wound edges, the group irradiated with low-intensity laser and the group treated with both laser and vitamin C had already partially formed epidermis, with a more organized underlying connective tissue and less evident inflammatory process. The group treated with laser alone obtained a higher concentration of type I collagen fibers and the group with the highest amount of type III collagen fibers was the one treated with the association of vitamin C and laser.

Conclusion: The present findings suggest that in spite of all treatments being effective in the repair of skin wounds compared to the control group, the isolated use of low-intensity therapy laser and its combined use with topical vitamin C showed the most favorable results, indicating that those could be further used for the treatment of skin wounds.

## Introduction

Ascorbic acid (vitamin C) is directly linked to skin healing due to its important role in the formation of collagen [1]. It has extremely important biological and metabolic functions, particularly with regard to its role in the biosynthesis of connective tissue, participating as a cofactor in the hydroxylation of proline and lysine, an essential reaction for collagen maturation and consequent resistance of wounds to tension [2]. In addition, ascorbic acid participates in cellular redox processes, in the synthesis of catecholamines and in the stimulation of cell proliferation [3].

Wound healing occurs through the contraction of the wound that is provided by the presence of myofibroblasts, a specialized form of fibroblast with contractile capacity [4], which also have the secretory function of elastin and collagen. It is reported that topical vitamin C increases the level of mRNA in collagen I and III, its conversion enzymes, and the tissue inhibitor of type I matrix metalloproteins in the human dermis [5].

The effects of laser therapy on wound healing have been reported by many studies [6-10]. Low-intensity laser therapy has been used in the treatment of wounds by accelerating the healing process and increasing cellular metabolism [11] as well as modulating both local and systemic immune responses [12]. Amongst the various therapies proposed for the treatment of wounds, the low-intensity laser stands out, and its use allows the acceleration of the wound healing process via growth factors [13-19] and by decreasing the inflammatory reaction [20]. The laser stimulation of fibroblasts during healing occurs through the maintenance of mitotic activity in the late period of healing [20], in which it has been demonstrated that low-intensity laser preferentially stimulates quiescent cells in detriment of those in activity [12].

Although demonstrating good outcomes in wound healing with low-intensity laser therapy [6-10], studies have not yet reported the correlation between laser and concomitant use of vitamin C. Therefore, the purpose of this study was to investigate wound healing by histologically evaluating tissue samples treated with vitamin C, laser and association of vitamin C and laser compared to a control group.

## Materials and methods

Experimental procedures were authorized and performed according to the protocols established by the Animal Use Ethics Committee of Anhanguera-Uniderp University, which are based on the Guide for the Care and Use of Laboratory Animals [21].

For the development of this study, 48 male rats (*Rattus norvegicus*, albinus, Wistar) were used, with weight varying between 250 and 300 g. The animals came from the bioterium of Anhanguera-Uniderp University, healthy and in satisfactory systemic conditions to be submitted to operative procedures. The rats were kept in cages, with four animals in each, fed with balanced Nuvital food and water "ad libitum" before and throughout the experimental period, except for 12 hours after the surgical procedure.

After anesthesia with sodium pentobarbital (10 mg/kg) intraperitoneally (IP), trichotomy of the dorsal region was performed in all animals and antisepsis of the entire area was performed with colorless Merthiolate® (Hypera S/A, Sao Paulo, Brazil). By using a punch, a circular area of skin of approximately 8 mm in diameter was removed in the dorsal region of the rats, more precisely in the middle portion of the median sagittal plane. The animals were divided into four groups of 12 animals each. In Group I (n=12) the wounds were treated with saline solution and were considered as a control group; Group II (n=12) received topical application of vitamin C (eight drops of Cewin®, Sanofi-Aventis, Sao Paulo, Brazil); Group III (n=12) was treated with low-intensity laser; and Group IV (n=12) received both topical application of vitamin C and low-intensity laser therapy.

The laser used was a DMC® model Photon Laser III (DMC Equipamentos Ltda, Sao Carlos, Brazil), with a power of 100 mW (power density of 3.58 W/cm2), a beam area of 0.028 cm², and a wavelength of 660 nm, active medium of Indian Phosphide-Gallium-Aluminum (InGaAlP) with energy density of 3 J/cm2 and time of 40 s per point. The wounds on the dorsum of the rats were irradiated once a day with dosage in joules per square centimeter (J/cm²) in five points of the wound, with the first application right after the injury was performed. On days 3 and 7 following the treatment of the wounds, samples of skin were obtained. Fixation, sample processing, inclusion, microtomy procedure, and histological analysis were performed on days 3 and 7.

The samples of skin were fixed in 10% buffered formaldehyde, for a minimum period of 24 hours, and then underwent the laboratory processes for inclusion in paraffin, being oriented in order to allow cross-sections with 5 µm of thickness. The sections were stained by the techniques of hematoxylin and eosin, picrosirius hematoxylin and Masson's trichrome, for histological analysis through optical microscopy. Tukey statistical analysis was used with 5% significance.

## Results

In both control and experimental groups, the inflammatory process, the presence of fibrinoid crust, epithelialization, and vascularization were analyzed.

**Figure 1:**
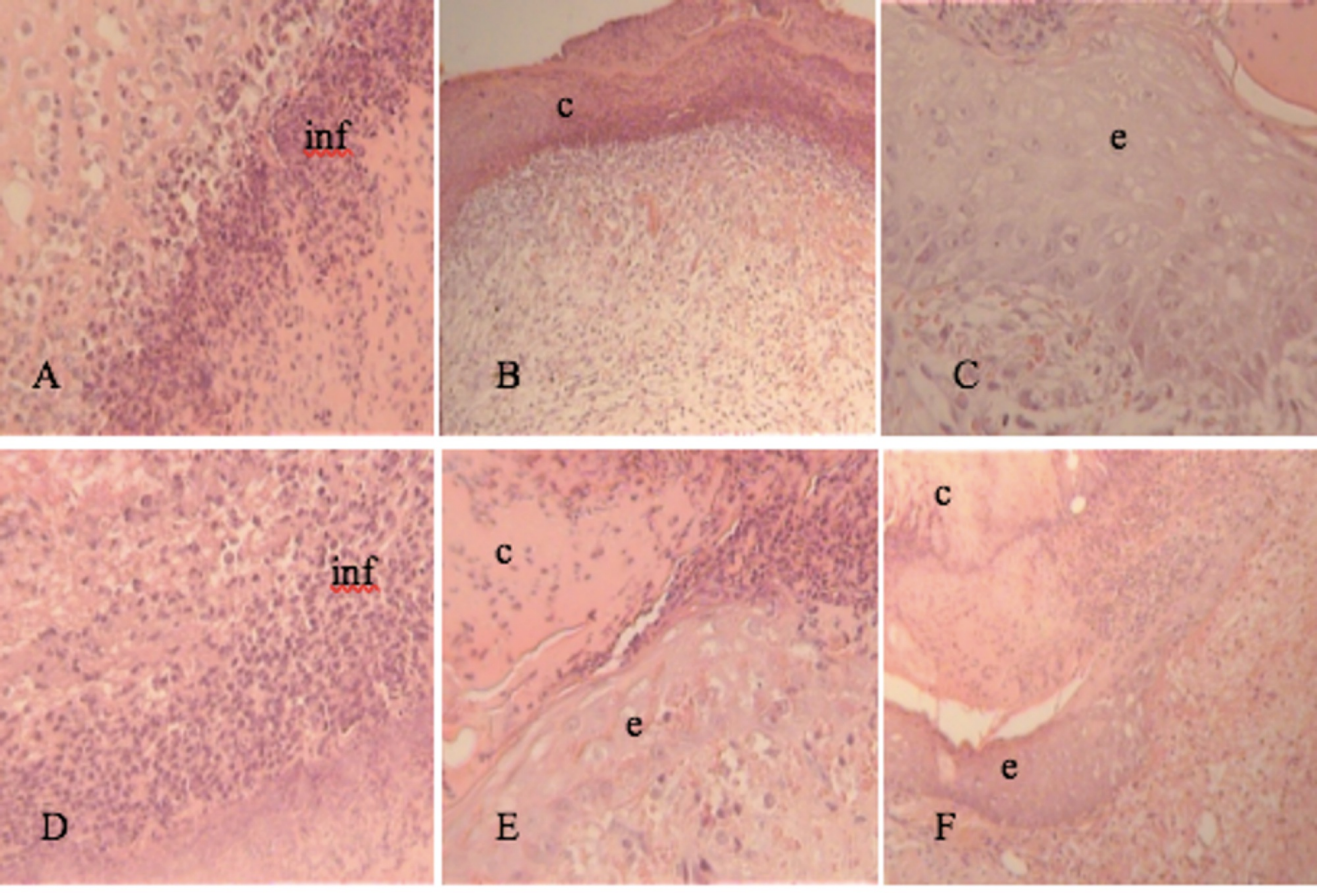
Tissue section on day 3. H&E stain 20-40x. A, B, C: Group I (Control Group) D, E, F: Group II (Vitamin C) inf, inflammation; c, crust; e, epithelium

**Figure 2:**
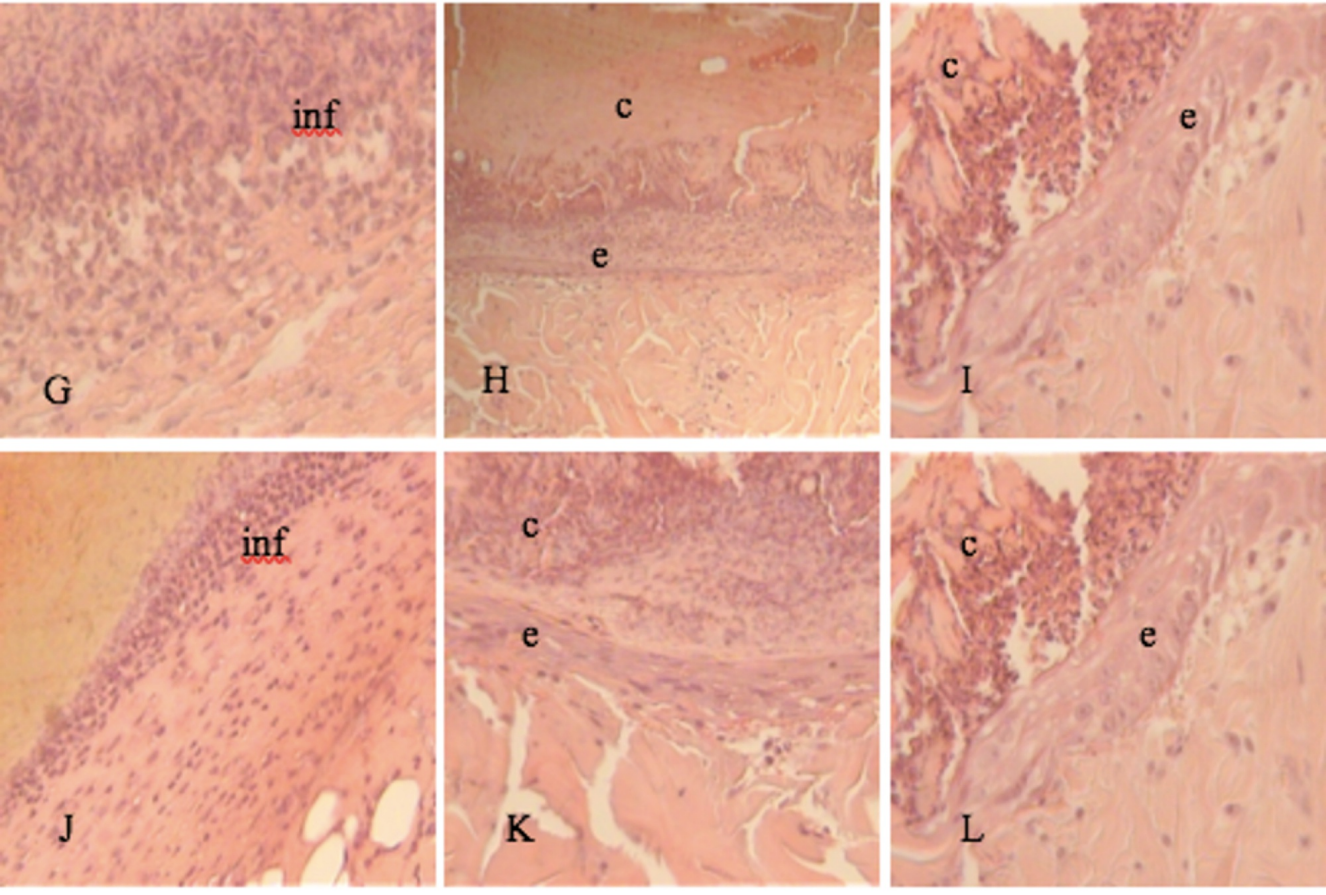
Tissue section on day 3. H&E stain 20-40x. G, H, I: Group III (Laser) J, K, L: Group IV (Laser + Vitamin C) inf, inflammation; c, crust; e, epithelium

At three days, the inflammatory process consisted mainly of neutrophils, which was more evident in groups I and II (Figure 1A,D). Above the skin wound, all samples showed fibrinoid crust and the beginning of an epithelium bud below this crust (Figure 1B,E; Figure 2H,K). In Group IV, small bundles of collagen fibers below the fibrinoid crust were observed (Figure 2L).

**Figure 3:**
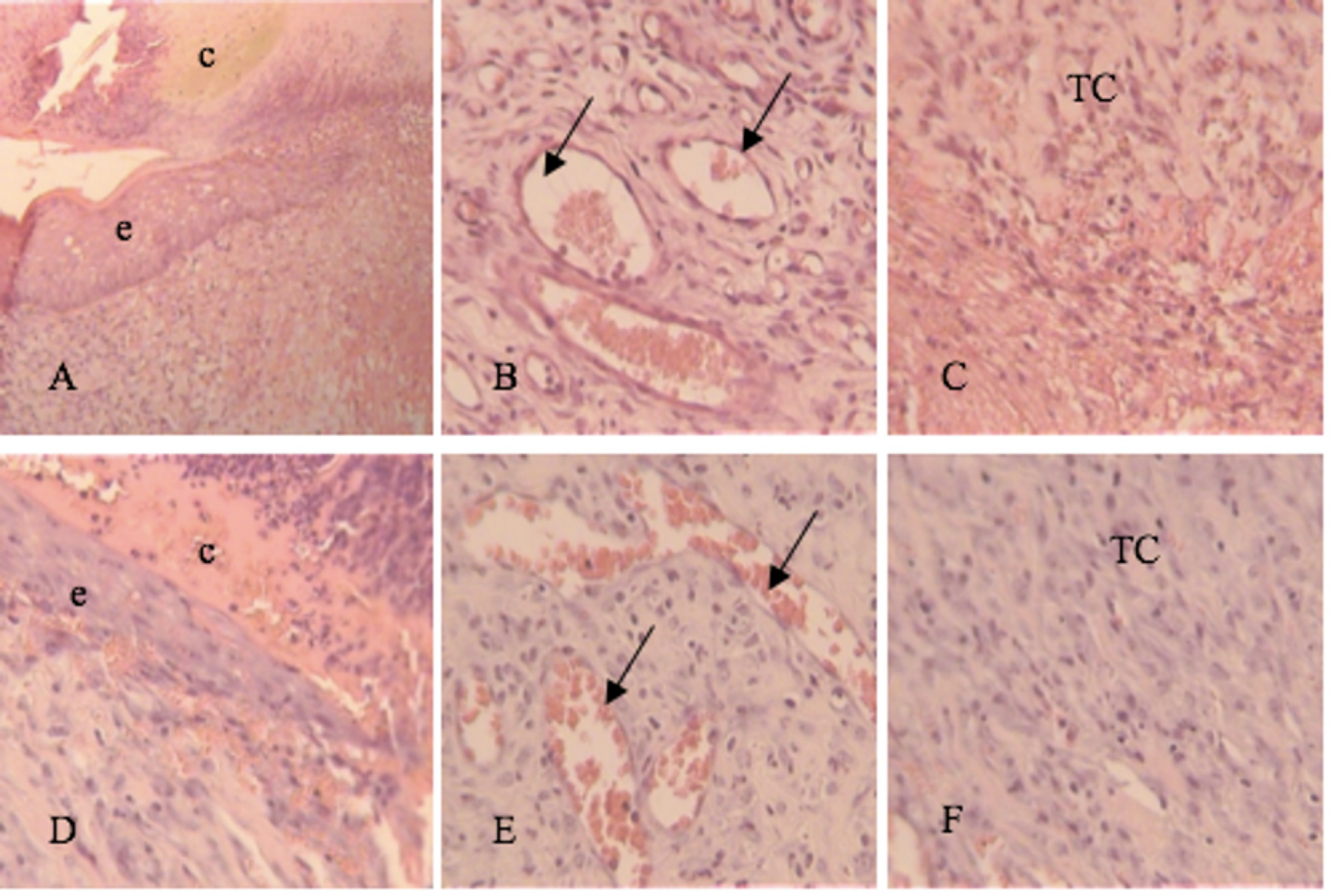
Tissue section on day 7. H&E stain 20-40x. A, B, C: Group I (Control Group) D, E, F: Group II (Vitamin C) c, crust; e, epithelium; TC, connective tissue; arrow, blood vessels

**Figure 4:**
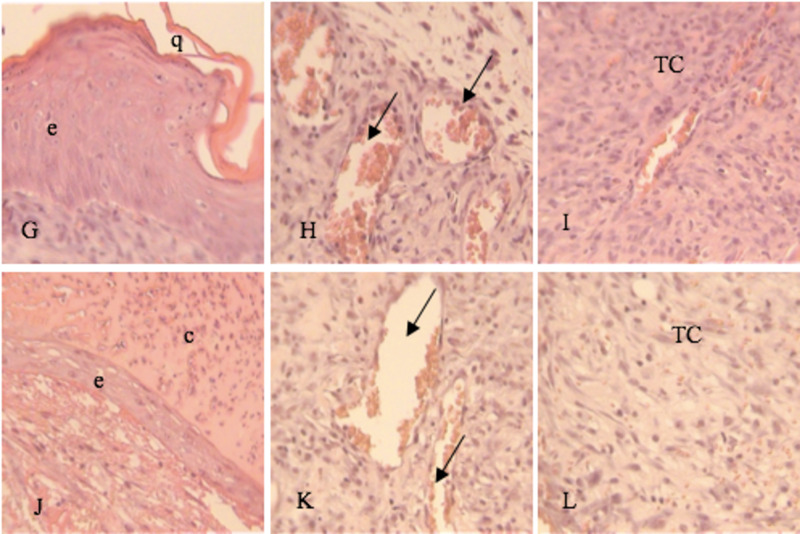
Tissue section on day 7. H&E stain 20-40x. G, H, I: Group III (Laser) J, K, L: Group IV (Laser+Vitamin C) c, crust; e, epithelium; q, keratin; TC, connective tissue; arrow, blood vessels

At seven days, epithelialization, vascularization, and the connective tissue below the epithelium were also analyzed. In Groups I and II it was shown a slight epithelial proliferation near the wound edges with presence of fibrinoid crust in some points of the wound (Figure 3A,D) whilst in Groups III and IV a partially formed epidermis was observed with a more robust crust (Figure 4G,J).

The connective tissue had a moderate number of fibroblasts alongside some macrophages and lymphocytes in the control group (Figure 3A), whereas in the other groups, the presence of a more organized underlying connective tissue was observed, with fibroblasts arranged parallelly to the wound surface (Figure 3F; Figure 4I,L). The formation of new vessels was the same in the four groups (Figure 3B,E; Figure 4H,K).

**Figure 5:**
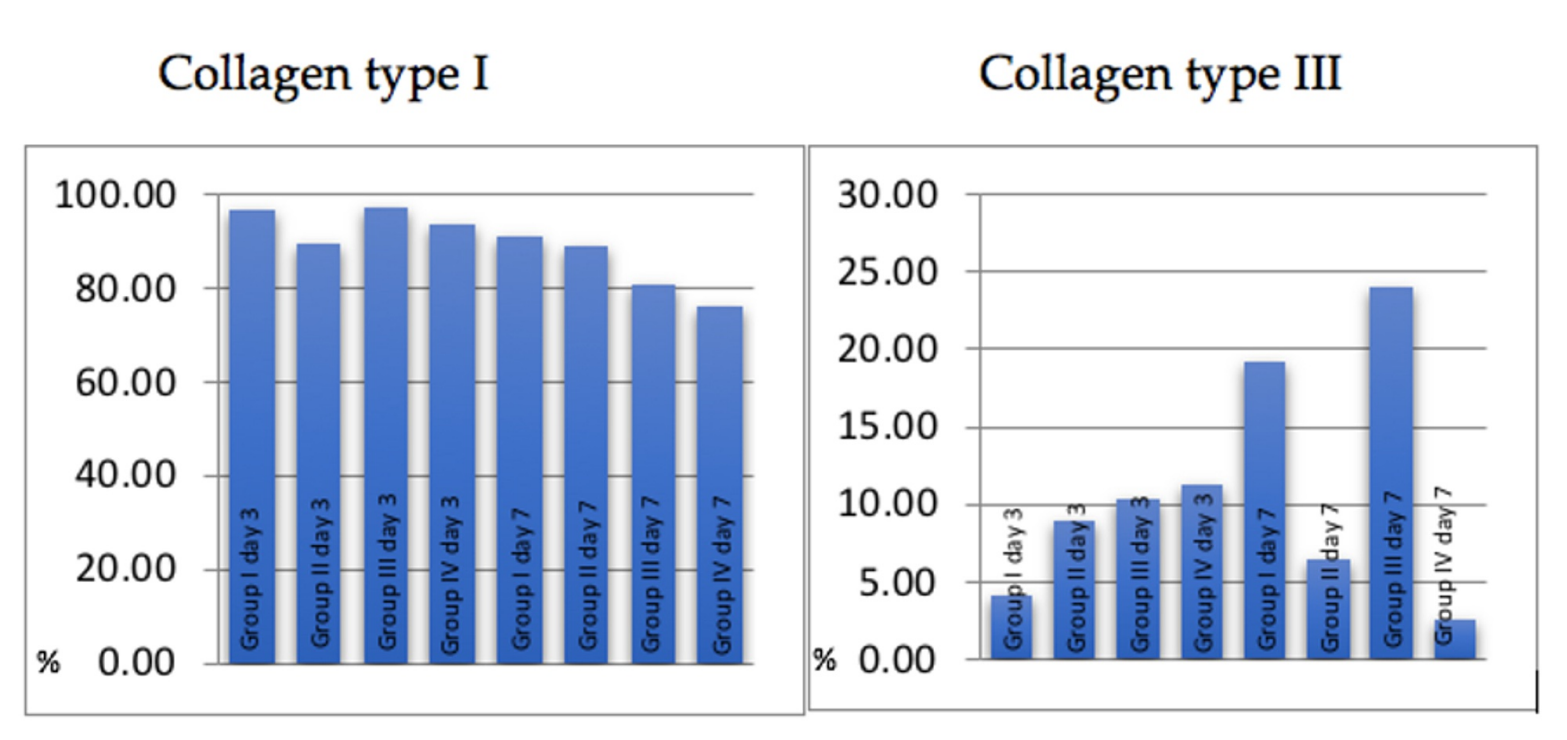
Concentration of collagen I and III using the picrosirius-hematoxylin histochemical method. From left to right: Group I day 3; Group II day 3; Group III day 3; Group IV day 3; Group I day 7; Group II day 7; Group III day 7; Group IV day 7 Tukey statistical analysis was used with 5% significance.

As observed in the analysis above (Figure 5), on day 3 Group III (treated with low-intensity laser alone) obtained a higher concentration of type I collagen fibers, but very close values were also achieved by the control group (I) and those treated only with vitamin C (II). As a result, it was observed that on day 3 there were no major differences between the group that received the effective treatment and the one that corresponded to the control group (p>0.05).

On day 7, the control group (I) excelled in the amount of type I collagen fibers, subsequently followed by the groups II, III, and IV. In view of this result, we observed that in all groups, except for those treated with vitamin C, there was a decrease in the amount of collagen I fibers, with the greatest decline in the group treated with laser.

With regard to the analysis of type III collagen fibers, on day 3 the highest amount of collagen fibers was found in the association of vitamin C with laser, followed by the group treated only with the laser, then vitamin C and control groups. On day 7, the group only treated with laser had a greater amount of type III fibers, followed by the control group and the group treated only with vitamin C. The group treated with vitamin C and laser showed the least amount of type III collagen fibers.

## Discussion

The present study aimed at verifying the effect of low-intensity laser therapy combined with vitamin C on the rate of wound healing, as both consist of therapeutic modalities that have been used in the healing process of wounds. Previous studies have demonstrated that both vitamin C and low-intensity laser therapy accelerate and facilitate wound healing [1, 5-6, 22-23].

The main function of collagen is to act as a scaffold in the connective tissue [24], especially in its type I, II, and III forms. During early process of wound healing, type III lays down first, and type I increases as scar is formed and remodeled [25]. This contributes to increasing tensile strength of the wound. Our study observed that after three days, concentrations of type III collagen were higher in the rats treated with an association of vitamin C and low-intensity laser, when compared to the other groups.

Several studies have analyzed the effects of low-intensity laser therapy [26-28] on skin wounds and some have looked into the mechanisms by which vitamin C aids skin healing [29-30], however, not many have used a combination of both treatments to evaluate why this combination therapy may be working better than individually. Therefore, this study opens potential scope for further research.

## Conclusions

From the results obtained through the histological analysis of the control and treatment groups on days 3 and 7 after the skin punch and wound treatment, it was observed that despite cutaneous repair in both groups, the presence of a milder inflammatory reaction, crust formation, epithelialization, and connective tissue formation was higher on the treatment groups when compared to the control group.

In addition, the rats whose wounds were treated with low-intensity laser and concurrent topical application of vitamin C showed a healing enhancing effect, with a less evident inflammatory process (after three days), partially formed epidermis, as well as the presence of connective tissue below a more organized epithelium, with fibroblasts arranged parallelly to the wound surface (after seven days). It is possible that due to the association of two factors that accelerate the wound repair process, by acting on the formation of collagen and increasing cellular metabolism, thus influencing the skin wound repair process, in a more satisfactory way when compared to all other groups in treatment.

Therefore, our results suggest that the association of low-intensity laser therapy and topical application of vitamin C could be used as a therapy modality for wound healing and could be further used as a clinical approach for treating cutaneous wounds.
